# HSP72 Protects Cells from ER Stress-induced Apoptosis via Enhancement of IRE1α-XBP1 Signaling through a Physical Interaction

**DOI:** 10.1371/journal.pbio.1000410

**Published:** 2010-07-06

**Authors:** Sanjeev Gupta, Ayswaria Deepti, Shane Deegan, Fernanda Lisbona, Claudio Hetz, Afshin Samali

**Affiliations:** 1 Apoptosis Research Centre, School of Natural Sciences, NUI Galway, Galway, Ireland; 2 Institute of Biomedical Sciences, FONDAP Center for Molecular Studies of the Cell, University of Chile, Santiago, Chile; Scripps Research Institute, United States of America

## Abstract

Endoplasmic reticulum (ER) stress is a feature of secretory cells and of many diseases including cancer, neurodegeneration, and diabetes. Adaptation to ER stress depends on the activation of a signal transduction pathway known as the unfolded protein response (UPR). Enhanced expression of Hsp72 has been shown to reduce tissue injury in response to stress stimuli and improve cell survival in experimental models of stroke, sepsis, renal failure, and myocardial ischemia. Hsp72 inhibits several features of the intrinsic apoptotic pathway. However, the molecular mechanisms by which Hsp72 expression inhibits ER stress-induced apoptosis are not clearly understood. Here we show that Hsp72 enhances cell survival under ER stress conditions. The UPR signals through the sensor IRE1α, which controls the splicing of the mRNA encoding the transcription factor XBP1. We show that Hsp72 enhances XBP1 mRNA splicing and expression of its target genes, associated with attenuated apoptosis under ER stress conditions. Inhibition of XBP1 mRNA splicing either by dominant negative IRE1α or by knocking down XBP1 specifically abrogated the inhibition of ER stress-induced apoptosis by Hsp72. Regulation of the UPR was associated with the formation of a stable protein complex between Hsp72 and the cytosolic domain of IRE1α. Finally, Hsp72 enhanced the RNase activity of recombinant IRE1α in vitro, suggesting a direct regulation. Our data show that binding of Hsp72 to IRE1α enhances IRE1α/XBP1 signaling at the ER and inhibits ER stress-induced apoptosis. These results provide a physical connection between cytosolic chaperones and the ER stress response.

## Abstract 2

The cytosolic chaperone Hsp72 directly modulates stress sensing in response to the accumulation of unfolded proteins in the endoplasmic reticulum and promotes cell survival.

## Author Summary

The endoplasmic reticulum (ER) is responsible for production and folding of secreted proteins. When the protein folding machinery cannot keep up with demand, misfolded proteins accumulate, leading to a state of ER stress that contributes to diseases such as cancer, neurodegeneration, diabetes, and myocardial infarct. The unfolded protein response (UPR) is an intracellular signaling network activated in response to ER stress. It initially tries to restore normal ER homeostasis, but if the damage is too severe cell death pathways mediated by cytosolic and mitochondrial proteins are activated. The molecular mechanisms involved in the transition of the UPR from a protective to an apoptotic phase are unclear. IRE1α is an ER membrane protein that acts as a sensor of ER stress. A number of proteins can interact with IRE1α to regulate its function, which includes an RNase activity responsible for inducing the unconventional splicing of the transcript for a downstream signaling protein called XBP-1. Here, we report that Hsp72, a stress-inducible cytosolic molecular chaperone, can bind to and enhance the RNase activity of IRE1α, providing an important molecular link between the heat shock response and the ER stress response. Importantly, increased production of active XBP-1 was necessary for Hsp72 to exert its prosurvival effect under conditions of ER stress. Our results suggest a mechanism whereby Hsp72 overexpression helps cells adapt to long-term ER stress in vivo by enhancing the pro-survival effects of the IRE1α/XBP1 branch of the UPR.

## Introduction

The human Hsp70 family consists of at least 12 members [1],[2]. Of these, the two best studied members are the constitutive or cognate Hsp70 (Hsc70) and a stress inducible form of cytosolic Hsp70 (Hsp72). Hsc70 is constitutively and ubiquitously expressed in tissues and has a basic and essential function as molecular chaperone in the folding of proteins [1],[2]. The second is an inducible form, called Hsp72, which is expressed at low levels under normal conditions and its expression is induced upon exposure to environmental stress that causes protein misfolding in the cytosol, such as heat shock, exposure to heavy metals, anoxia, and ischemia [1],[2]. Hsp72 has strong cytoprotective effects and functions as a molecular chaperone in protein folding, transport, and degradation. Moreover, the cytoprotective effect of Hsp72 is also related to its ability to inhibit apoptosis [3],[4]. Hsp72 has been shown to inhibit apoptosis by several distinct mechanisms [3],[5],[6]. It can prevent the formation of an active apoptosome by binding directly to Apaf-1, in in vitro conditions [7],[8]. Additionally, it has been shown that Hsp72 functions upstream of the caspase cascade by inhibiting the release of cytochrome *c* from the mitochondria [9],[10],[11]. Inhibition of cytochrome *c* release may be achieved by the ability of Hsp72 to prevent Bax translocation into the mitochondrial membrane in response to stress [9],[10],[11]. It has also been shown that Hsp72 inhibits apoptosis by suppressing JNK, a stress-activated protein kinase, thereby blocking an early component of a stress-induced apoptotic pathway [12]. Further, it has been shown that Hsp72 binds to apoptosis-inducing factor (AIF), another apoptogenic factor released from the mitochondria, thereby preventing the chromatin condensation and cell death that result from AIF [13],[14],[15].

Physiological or pathological processes that disrupt protein folding in the endoplasmic reticulum (ER) lead to ER stress and trigger a set of signaling pathways termed the unfolded protein response (UPR) [16]. This concerted and complex cellular response transmits information about the protein-folding status in the ER lumen to the cytosol and nucleus to increase protein-folding capacity [17],[18]. However, cells undergo apoptosis if these mechanisms of cellular adaptation are unable to alleviate the stress [19]. The three major transmembrane sensors of ER stress in metazoans are IRE1α (inositol requiring 1; ERN1, endoplasmic reticulum-to-nucleus signaling 1), PERK [double-stranded RNA-activated protein kinase (PKR)-like ER kinase; PEK, pancreatic eukaryotic initiation factor 2α kinase; EIF2AK3], and ATF6 (activating transcription factor 6) [17],[18]. IRE1α, the prototype ER stress sensor, is evolutionarily conserved from yeast to humans and the cytoprotective output of IRE1α is present across all eukaryotes. IRE1α is a Ser/Thr protein kinase and endoribonuclease that, upon activation, initiates the unconventional splicing of the X-box binding protein (XBP1) mRNA [20]. The spliced XBP1 form is a highly active transcription factor and one of the key regulators of ER folding capacity [21],[22]. In response to ER stress, IRE1α splices a 26 nucleotide long intron of unspliced XBP1 mRNA (XBP1u), generating an active and stable transcription factor XBP1s. XBP1s regulates several UPR target genes including ER chaperones (BIP/GRP78, ERdj4, ERdj5, HEDJ, GRP58, and PDIP5), ERAD components (EDEM, HERP, and p58^IPK^), transcription factors (CHOP and XBP1), and other proteins related to the secretory pathway [23].

The activation of IRE1α is regulated by a complex protein platform at the ER membrane, known as the UPRosome [24],[25]. BAX and BAK form a protein complex with the cytosolic domain of IRE1α, which requires their conserved BH1 and BH3 domains [26]. Similarly, ASK1-interacting protein 1 (AIP1) has been shown to associate with IRE1α and to enhance the dimerization of IRE1α, suggesting a direct role for AIP1 in regulating IRE1α activity [27]. We have shown that Bax inhibitor-1 (BI-1) also binds to IRE1α and has an inhibitory effect on IRE1α signaling [28],[29]. Furthermore, ER localized Protein Tyrosine Phosphatase 1B (PTP 1B) has been show to potentiate the IRE1α signaling pathway, however its interaction with IRE1α has not been determined [30]. It has been proposed that binding of anti- and pro-apoptotic proteins to IRE1α controls the amplitude of IRE1α signaling and determines cell fate during conditions of ER stress.

The cytoprotective role of Hsp72 has been demonstrated in many tissues and its role as a neuroprotectant has been demonstrated in vitro and in animal models of neuronal degeneration in vivo [31],[32],[33]. Transgenic mice overexpressing the *hsp72* gene show significant protection during focal cerebral ischemia [34],[35]. Further, injection of a vector carrying the *hsp72* gene into the rat hippocampal CA1 region provides protection to cells in the vicinity of the injection site following 10 min of global ischemia [36]. Despite a large number of studies demonstrating neuroprotection by the chaperone Hsp72, in both animal [31],[34],[35] and cell culture models of ischemia [33],[37], the mechanisms of protection are poorly understood. The presence of chronic ER stress has been extensively described in neurodegenerative conditions linked to protein misfolding and aggregation, including Amyotrophic Lateral Sclerosis (ALS), prion-related disorders, and conditions such as Parkinson's, Huntington's, and Alzheimer's disease. We reasoned that Hsp72 may provide cytoprotection by modulation of UPR signaling pathways emanating from the ER membrane. Here we have evaluated the effect of Hsp72 on UPR signaling. Overall our results identify Hsp72 as a new component of UPRosome where binding of Hsp72 to IRE1α enhances IRE1α-XBP1 signaling at the ER, promoting adaptation to ER stress and cell survival.

## Results

### Hsp72 Expression Inhibits ER Stress-Induced Apoptosis Upstream of Mitochondria

**Figure 1:**
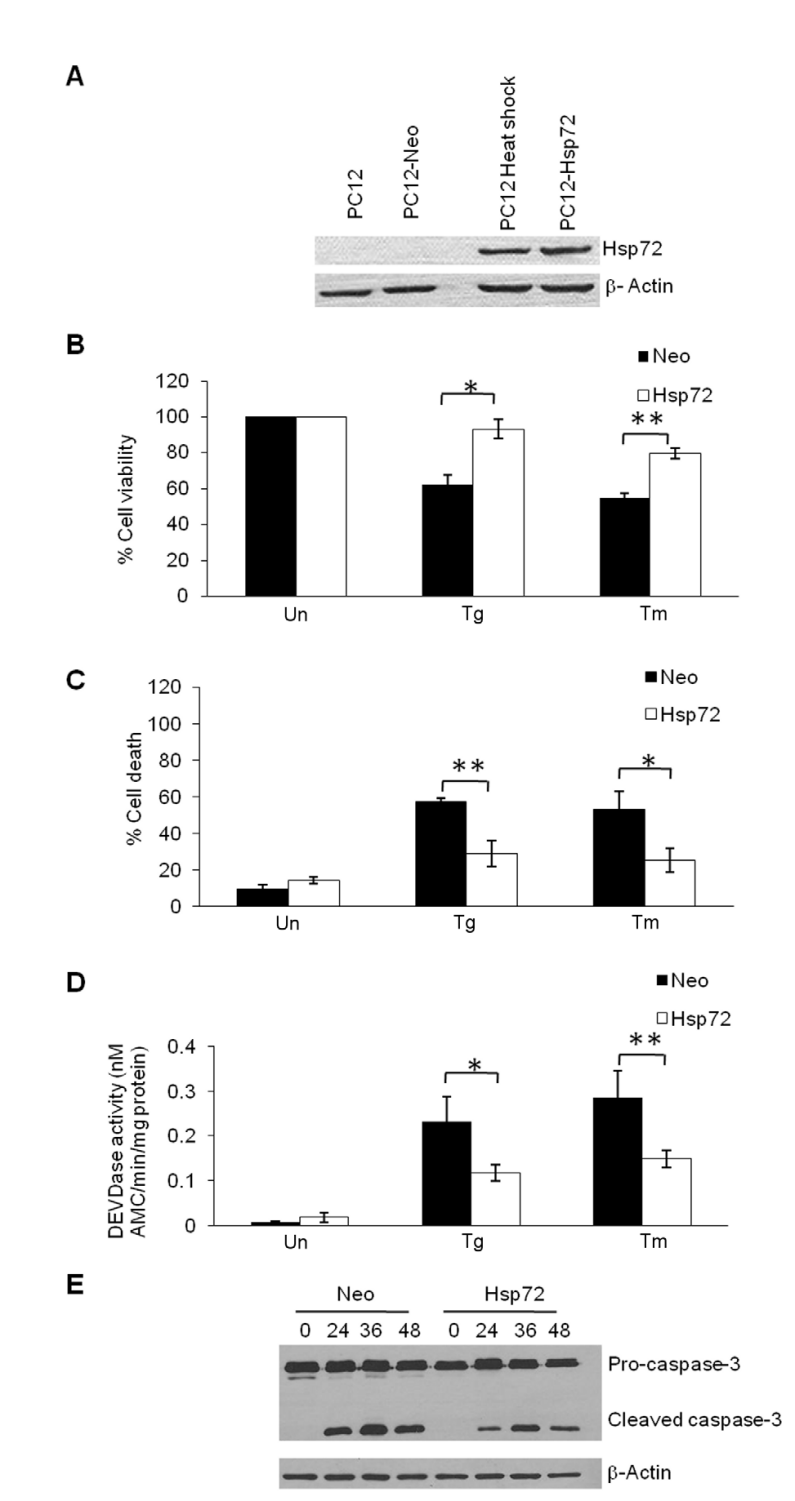
Hsp72 protects PC12 from apoptosis induced by ER stress. (A) Immunoblotting of total protein from indicated cells was performed using antibodies against Hsp72 and β-actin. (B) The control (Neo) and Hsp72 expressing (Hsp72) PC12 cells were either untreated (Un) or treated with (0.25 µM) thapsigargin (Tg) or (2 µg/ml) tunicamicin (Tm) for 48 h. Reduction in cell viability was determined by MTT assay. Average and error bars represent mean ± SD from three independent experiments performed in triplicate. (C) Cells were treated as in (B), and apoptosis was determined with annexin-V/PI staining followed by FACS analysis. Percentages of cells positive for both annexin-V and PI are shown. Average and error bars represent mean ± SD from three independent experiments. (D) Cells were treated as in (B), and DEVDase activity was measured as described in Materials and Methods. Average and error bars represent mean ± SD from four independent experiments performed in duplicate. (E) The control (Neo) and Hsp72 expressing (Hsp72) PC12 cells were treated with (0.25 µM) Tg for the indicated time and Western blotting of total protein was performed using antibodies against caspase-3 and β-actin. * indicates a statistical significance between Neo and Hsp72 cells; *p*<0.05. ** indicates a statistical significance between Neo and Hsp72 cells; *p*<0.005.

Neuroprotective effects of Hsp72 overexpression have been reported in numerous studies during ischemia-like conditions in neuronal cells [15],[31],[32]. To assess the effect of Hsp72 expression on ER stress-induced apoptosis, we generated stable clones of PC12 cells expressing the inducible form of Hsp70 (Hsp72). The level of Hsp72 expression in PC12 cells used in this study was within the normal physiological range, because ectopic Hsp72 expression is comparable to the level of Hsp72 induced during thermotolerance in PC12 cells (Figure 1A). For induction of thermotolerance, cells were subjected to 1 h of heat shock at 42°C±0.5°C and processed after a 6 h recovery at 37°C. To determine the effect of Hsp72 expression on ER stress-induced apoptosis, control (Neo) and Hsp72-expressing (Hsp72) PC12 cells were treated with either 0.25 µM thapsigargin or 1 µg/ml tunicamycin for 48 h. We observed that Hsp72 expression partially protected PC12 cells from ER stress-induced cell death (Figure 1B, C). ER stress-induced caspase activity was found to be significantly reduced in Hsp72 cells as compared with Neo cells (Figure 1D). In agreement with reduced caspase activity, Hsp72 cells showed reduced processing of pro-caspase-3 to active caspase-3 (Figure 1E). These results suggest that caspase activity is required for ER stress-induced apoptosis and that Hsp72 can inhibit the ability of the cell to activate the caspase cascade.

**Figure 2:**
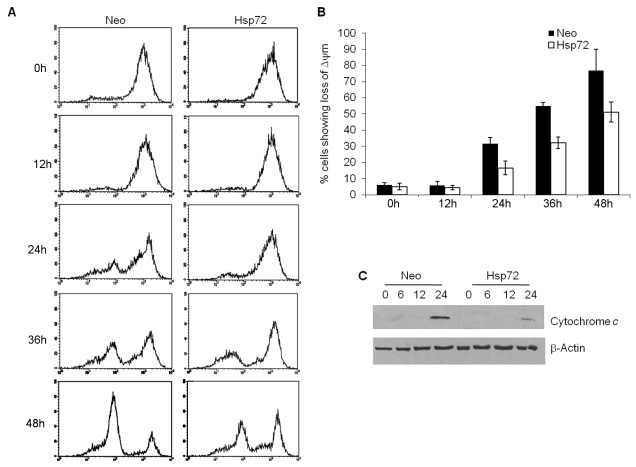
Hsp72 prevents ER stress-induced loss of mitochondrial membrane potential and cytochrome *c* release. The control (Neo) and Hsp72 expressing (Hsp72) PC12 cells were treated with (0.25 µM) Tg for the indicated time points. (A) Mitochondrial membrane potential was assessed by TMRE staining and flow cytometry. A representative image of three independent experiments is shown. (B) Following treatment, cells were incubated with TMRE (100 nM). Mitochondrial membrane potential was monitored by measuring the fluorescence intensity at 582 nm (FL2). Average and error bars represent mean ± SD from three independent experiments. (C) Cytosolic extracts were prepared as described in Materials and Methods and resolved by SDS-PAGE followed by Western blotting using antibodies against cytochrome *c* and β-actin.

The loss of mitochondrial membrane potential (ΔΨm) and MOMP is a hallmark of apoptosis [38],[39]. Previous studies have shown that Hsp72 inhibits apoptosis by preventing mitochondrial outer membrane permeabilization and cytochrome *c* release [10],[11]. Next we evaluated the effect of Hsp72 on the dissipation of ΔΨm and release of cytochrome *c* to the cytosol upon exposure to ER stress stimuli. To quantify ΔΨm, TMRE, a potentiometric fluorescent dye that incorporates into mitochondria in a ΔΨm-dependent manner, was used. Cells were either left untreated or treated with 0.25 µM thapsigargin. The cells were then incubated with TMRE for 30 min and analyzed by a flow cytometer. A drop in ΔΨm was observed in Neo cells following thapsigargin treatment (Figure 2A). The expression of Hsp72 inhibited the loss of ΔΨm (Figure 2A). At 48 h, loss of ΔΨm was detected in 80%–90% of Neo cells treated with thapsigargin or tunicamycin, respectively (Figure 2B). However, at the same time point, thapsigargin or tunicamycin only induced loss of ΔΨm in 50% of the Hsp72 cells (Figure 2B). To further study the involvement of mitochondria in ER stress-induced cell death, we assessed the release of cytochrome *c* into the cytosol. Western blot analysis of the cytosolic extracts of cells showed that exposure of Neo cells to thapsigargin for 24 h triggered release of cytochrome *c* from mitochondria (Figure 2C). However, at the same time point, the release of cytochrome *c* induced by thapsigargin was significantly reduced in Hsp72 cells (Figure 2C). These results suggest that Hsp72 may be acting upstream of MOMP to inhibit ER stress-induced apoptosis.

### Hsp72 Expression Enhances XBP1 mRNA Splicing under ER Stress Conditions

**Figure 3:**
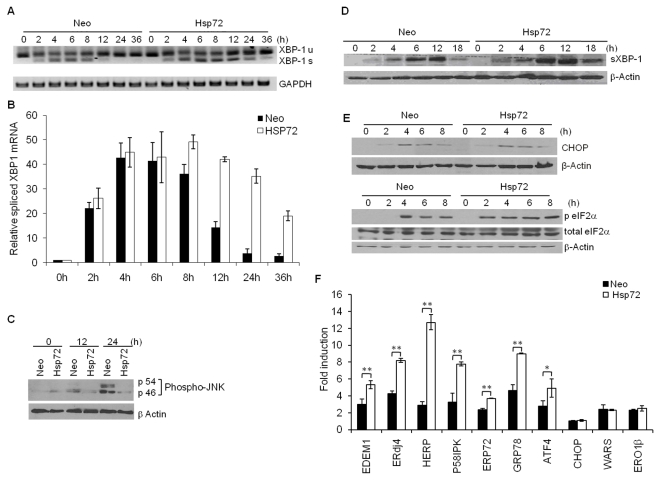
ER stress-induced activation of IRE1α/XBP1 axis is increased in Hsp72 expressing cells. (A) The control (Neo) and Hsp72 expressing (Hsp72) PC12 cells were treated with (0.1 µM) Tg for indicated time points. RT-PCR analysis of total RNA was performed to simultaneously detect both spliced and unspliced XBP1 mRNA and GAPDH. Size of PCR products: unspliced XBP1 = 289 bp, spliced XBP1 = 263 bp. The image is presented inverted for greater clarity. (B) In the experiment described in (A), XBP1 mRNA splicing was calculated after densitometric analysis of the XBP1s PCR products. Average and error bars represent mean ± SD from three independent experiments. (C–E) The control (Neo) and Hsp72 expressing (Hsp72) PC12 cells were treated with (0.25 µM) Tg for the indicated time points. (C) Immunoblotting of total protein was performed using antibodies against phospho-JNK and β-actin. (D) Immunoblotting of total protein was performed using antibodies against spliced XBP1 and β-actin. (E) Immunoblotting of total protein was performed using antibodies against CHOP, phospho-eIF-2α, total eIF-2α, and β-actin. (F) The control (Neo) and Hsp72 expressing (Hsp72) PC12 cells were treated with (0.1 µM) Tg for 12 h, and the expression level of indicated genes was quantified by real-time RT-PCR, normalizing against GAPDH. Average and error bars represent mean ± SD from two independent experiments performed in triplicate. * indicates a statistical significance between Neo and Hsp72 cells; *p*<0.05. ** indicates a statistical significance between Neo and Hsp72 cells; *p*<0.005.

Activation of the UPR and regulation of protein quality control is essential to restore cellular homeostasis and prevent ER stress-induced apoptosis [18],[19]. To investigate the possible regulation of the UPR by Hsp72, we compared the activation of IRE1α/XBP1 and PERK/CHOP axis in Neo and Hsp72 cells. First we determined the levels of XBP1 mRNA splicing by semi-quantitative RT-PCR and production of spliced XBP1 protein by Western blotting. Notably, upon treatment with thapsigargin Hsp72 cells displayed increased levels of the spliced XBP1 mRNA as compared to Neo cells, demonstrating a sustained signaling over time and late inactivation (Figure 3A,B). In agreement with the increased XBP1 mRNA splicing, enhanced expression of XBP1s protein was also observed in Hsp72 cells undergoing ER stress when compared with Neo cells (Figure 3D). Since JNK activation is also induced downstream of IRE1α activation, we next determined the effect of Hsp72 on JNK activation during ER stress signaling. Activation of JNK was detected by Western blotting with a phospho-specific antibody. ER stress-induced JNK phosphorylation was reduced in Hsp72 cells as compared to Neo cells (Figure 3C). Activation of the PERK/CHOP axis, a parallel pathway activated by ER stress, was also examined by measuring phosphorylation of eIF-2α, a direct target of PERK, and expression of CHOP. The level of ER stress-induced phosphorylation of eIF-2α and induction of CHOP was not significantly different in Hsp72 cells as compared to Neo cells, although Hsp72 cells showed slightly earlier kinetics in eIF-2α phosphorylation (Figure 3E). In conditions of ER stress, cellular adaptation is mediated by modulating the expression of a cohort of so-called UPR target genes. The IRE1α/XBP1 arm of the UPR specifically mediates the induction of specific target genes such as EDEM1, ERdj4, and P58^IPK^ [22],[40]. Analysis of gene expression profiles by quantitative RT-PCR revealed that induction of EDEM1, ERdj4, HERP, P58^IPK^, and GRP78 was significantly enhanced in Hsp72 cells as compared to Neo cells (Figure 3F). Taken together, these observations suggest that Hsp72 specifically regulates ER stress signaling through the modulation of the IRE1α/XBP1 axis of the UPR.

### Increased XBP1s Protein Is Required for Enhanced Cell Survival Induced by Hsp72 Under ER Stress Conditions

**Figure 4:**
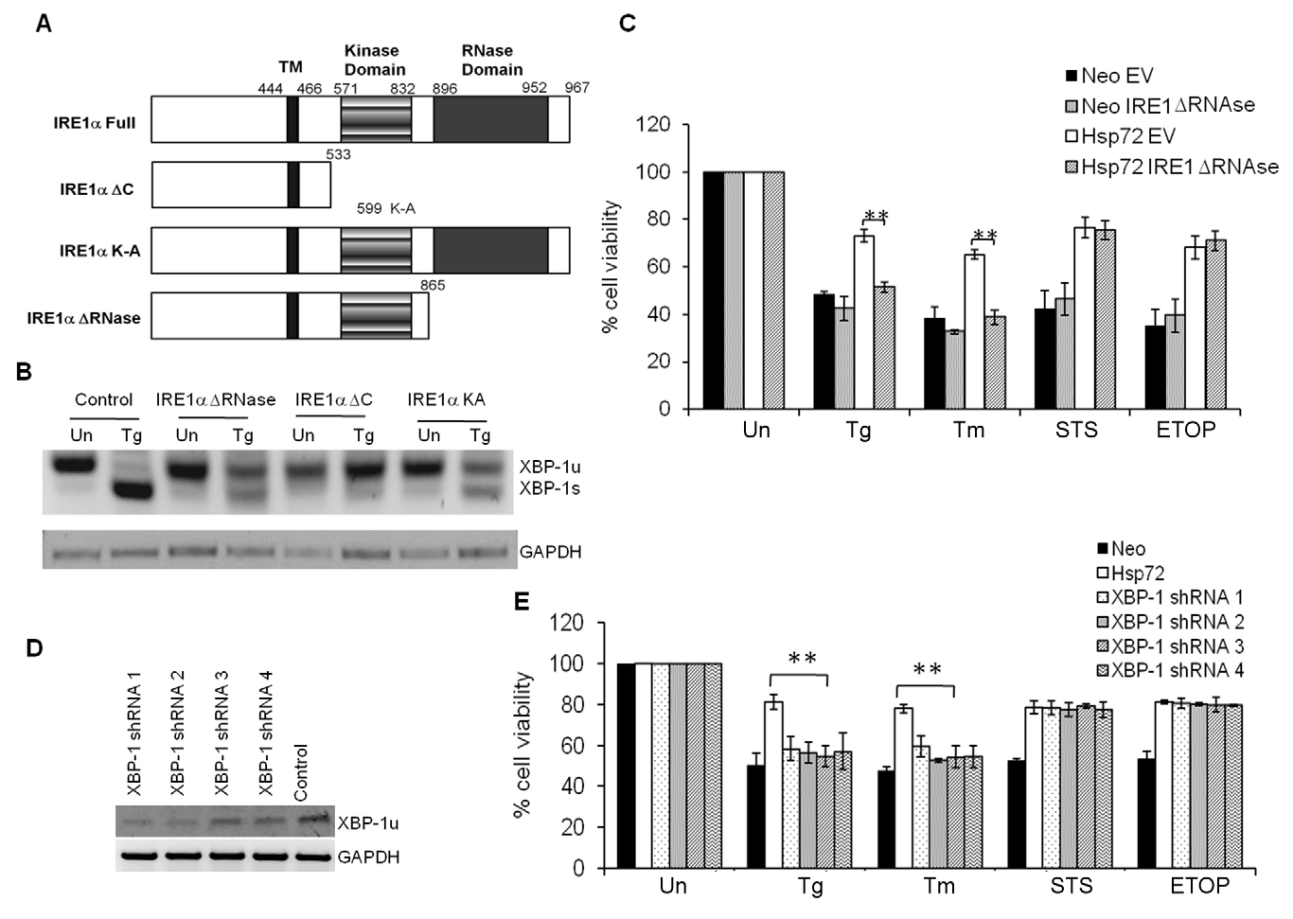
Increased production of spliced XBP1 contributes to cytoprotective function of Hsp72 against ER stress-induced apoptosis. (A) Schematic presentation of wild-type and mutant IRE1α plasmids. (B) PC12 cells were transfected with indicated IRE1α plasmids. 24 h post-transfection, cells were either untreated (Un) or treated with (0.25 µM) Tg for 6 h. RT-PCR analysis of total RNA was performed to simultaneously detect both spliced and unspliced XBP1 mRNA and GAPDH. The image is presented inverted for greater clarity. (C) pCMV.SPORT-βGAL was co-transfected with either pcDNA3.1 or IRE1α ΔRNase expression plasmid in control (Neo) or Hsp72 expressing (Hsp72) PC12 cells. 24 h post-transfection, cells were either left untreated (Un) or treated with (0.25 µM) Tg for 48 h, (2 µg/ml) Tm for 48 h, (150 nM) staurosporine (STS) for 16 h, or (25 µg/ml) etoposide (ETOP) for 24 h. The reduction in cell viability was determined by measuring the reduction in β-galactosidase activity after the drug treatments. Average and error bars represent mean ± SD from three independent experiments performed in triplicate. ** indicates a statistical significance between Hsp72 and Hsp72 plus IRE ΔRNase cells; *p*<0.005. (D) Hsp72 expressing PC12 cells were transduced with lentivirus expressing indicated XBP1 targeting shRNA. RT-PCR analysis of total RNA was performed to simultaneously detect unspliced XBP1 mRNA and GAPDH. The image is presented inverted for greater clarity. (E) The control (Neo), Hsp72 expressing (Hsp72), and Hsp72 cells expressing indicated shRNAs were either untreated (Un) or treated with (0.25 µM) Tg, (2 µg/ml) Tm, (150 nM) staurosporine (STS), or (25 µg/ml) etoposide (ETOP). The reduction in cell viability was determined by MTT assay. Average and error bars represent mean ± SD from three independent experiments performed in triplicate. ** indicates a statistical significance between Hsp72 and Hsp72 plus XBP1 shRNA cells; *p*<0.005.

Recently it has been shown that experimental prolonging of IRE1α signaling independent of ER stress can promote cell adaptation to protein folding stress and survival [9],[41]. Our data show that the ability of Hsp72 to inhibit ER stress-induced apoptosis correlates with enhanced production of spliced XBP1. To determine the role of XBP1s in the cytoprotective effects of Hsp72, we used a dominant negative mutant of IRE1α to compromise the production of spliced XBP1 and evaluated its effect on the protection mediated by Hsp72. Expression vectors for various mutants of IRE1α (IRE1α KA, IRE1α ΔC, and IRE1α ΔRNase) (Figure 4A) were transfected into PC12 cells, and the levels of XBP1 mRNA splicing were examined upon ER stress. We observed that the three mutants of IRE1α reduced ER stress-induced splicing of XBP1 as compared to control pcDNA transfected cells (Figure 4B). Further experiments were performed with the IRE1α ΔRNase because the compromised kinase domain in IRE1α KA or the lack of kinase domain in IRE1α ΔC may alter the downstream events mediated by the kinase domain of IRE1α in addition to abrogating its endoribonuclease activity. The effect of IRE1α ΔRNase on cell viability was determined by measuring β-galactosidase activity after treatment with ER stress-inducing agents thapsigargin and tunicamycin, and two other apoptosis-inducing agents that do not act through ER stress, staurosporine and etoposide [42]. The IRE1α ΔRNase mutant was co-transfected with β-galactosidase plasmid into Neo and Hsp72 cells and the reduction in reporter enzyme activity was used to determine whether a gene has a detrimental effect on cell survival [42]. We observed that IRE1α ΔRNase mutant specifically reversed the protective effect of Hsp72 on ER stress-induced apoptosis, but it did not affect the protection against etoposide and staurosporine (Figure 4C). To further confirm the role of increased XBP1s protein in the cytoprotective effects of Hsp72, we knocked down XBP1s levels by introducing XBP1 targeted shRNAs into Hsp72 cells and then assessed their effects on cell survival. We found that all four shRNAs were able to silence XBP1s expression to varying degrees (Figure 4D). Notably, the protective effect of Hsp72 during ER stress-induced apoptosis was abrogated in four independent subclones of Hsp72 cells expressing XBP1 targeted shRNAs (Figure 4E). These results suggest that all four XBP1 targeting shRNAs are able to neutralize the effect of Hsp72 overexpression on ER stress-induced production of spliced XBP1 and apoptosis. The knockdown of XBP1 did not alter the cytoprotective effects of Hsp72 on staurosporine- or etoposide-induced apoptosis (Figure 4E). Collectively, these results suggest that Hsp72 enhances survival under ER stress conditions possibly by upregulation of the adaptive responses initiated by the IRE1α/XBP1 branch of the UPR.

### Hsp72 Forms a Protein Complex with IRE1α

**Figure 5:**
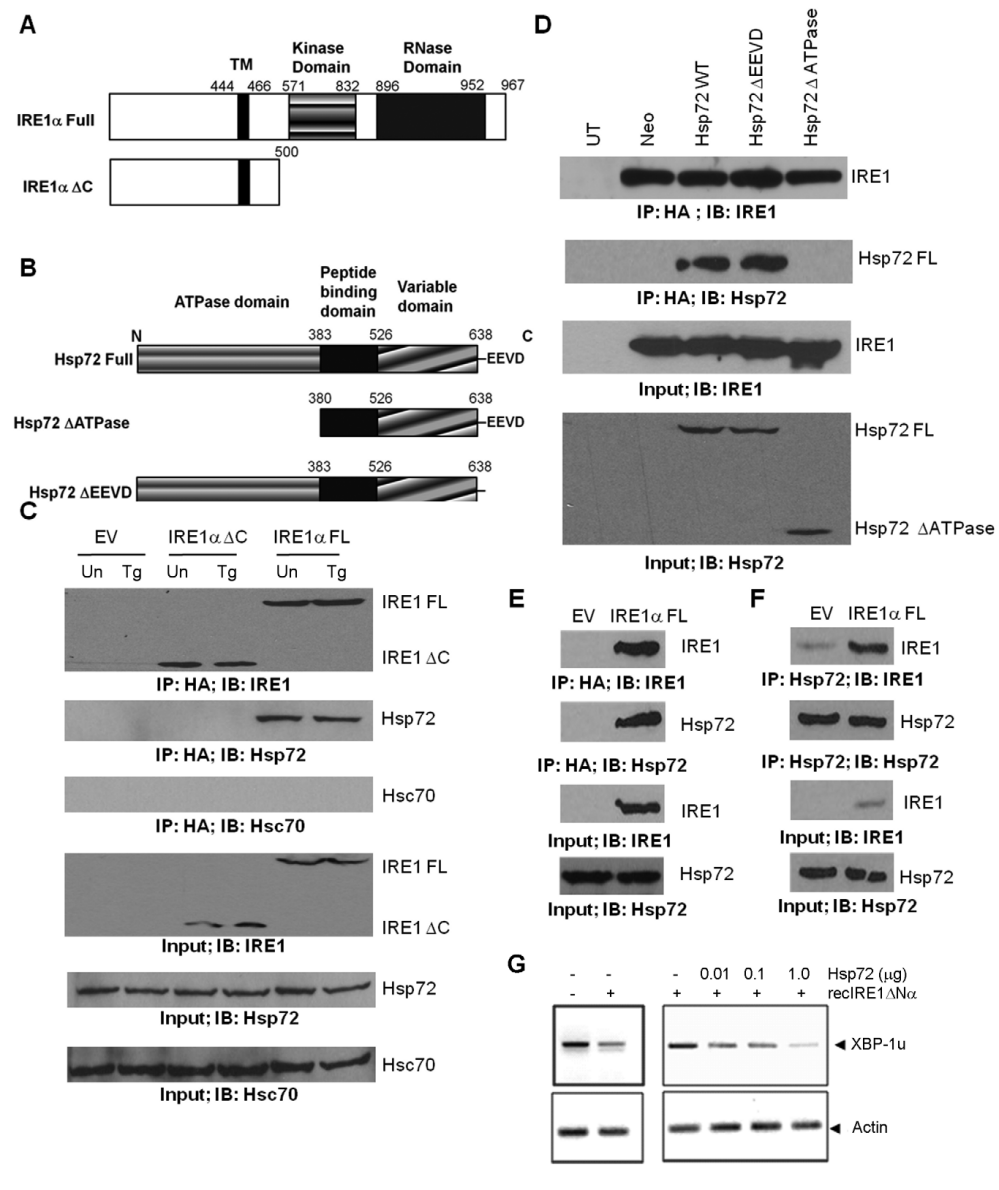
Hsp72 forms a protein complex with IRE1α, and the ATPase domain of Hsp72 is critical for IRE1α binding. (A) Schematic diagram for IRE1α structure domains and expression constructs. (B) Schematic diagram for Hsp72 structure domains and expression constructs. (C) Hsp72 expressing PC12 cells (Hsp72) were transfected with empty vector (EV) or expression vectors for IRE1α FL-HA or IRE1α ΔC-HA. After 24 h, cells were either untreated (Un) or treated with (0.25 µM) Tg for 12 h and then the co-precipitation of Hsp72 with IRE1α FL-HA or IRE1α ΔC-HA was evaluated by IP and Western blot. (D) PC12 cells expressing the indicated Hsp72 constructs were transfected with IRE1α FL-HA. Lysate from untransfected PC12 cells (UT) was used as a negative control. Co-precipitation of wild-type, ΔATPase, and ΔEEVD mutant of Hsp72 with IRE1α FL-HA was evaluated by IP and Western blot. (E) HEK 293 cells were transiently transfected with IRE1α FL-HA expression vector or empty vector (EV). After 48 h, IRE1α FL-HA was immunoprecipitated and its association with endogenous Hsp72 was assessed by Western blot. (F) Endogenous Hsp72 was immunoprecipitated from HEK 293 cells transiently transfected with IRE1α FL-HA expression vector or empty vector (EV), and its association with IRE1α was determined by Western blot analysis. Input: 5% of the total cell lysate used for IPs. (G) Indicated concentrations of recombinant Hsp72 was incubated with 1 µg recIRE1ΔNα for 1 h at 30°C in the presence of ATP, and 10 µg total mRNA. The ribonuclease activity of IRE1α was analyzed by RT-PCR by using regular XBP1 mRNA splicing primers, evidenced as decreased PCR product of the nonspliced fragment. Actin was monitored as control (lower panel). A representative of three independent experiments is shown.

To determine the mechanism by which Hsp72 regulates IRE1α activity, we first explored the possibility of a physical interaction between Hsp72 and IRE1α. For this purpose, Hsp72 cells were transfected with IRE1α FL-HA or IRE1α ΔC-HA (Figure 5A) and interaction of Hsp72-IRE1α was determined by co-immunoprecipitation assays. The Hsp72-IRE1α complex was detected in the absence of ER stress and required the cytosolic C-terminal region of IRE1α, which encodes the kinase and endoribonuclease domains (Figure 5C). Further, the interaction of Hsp72 with IRE1α was not altered in cells undergoing ER stress triggered by thapsigargin treatment (Figure 5C). Under similar conditions, Hsc70, the constitutive form of Hsp72 did not interact with IRE1α (Figure 5C). Hsp72 consists of three structural motifs: an N-terminal ATPase domain, a C-terminal substrate binding domain, and a C-terminal sequence EEVD (Figure 5B). Hsp72 function requires coordinated action of all three domains. To map the critical domain of Hsp72 required for IRE1α binding, we transfected IRE1α FL-HA into PC12 cells expressing Hsp72, ΔATPase-Hsp72, or ΔEEVD-Hsp72 (Figure 5B) and association of IRE1α with wild-type and mutant Hsp72 was determined by co-immunoprecipitation assays. We observed that wild-type Hsp72 and ΔEEVD-Hsp72 associated with IRE1α (Figure 5D). However, ΔATPase- Hsp72 failed to interact with IRE1α, demonstrating that the ATPase domain of Hsp72 is necessary for interaction of Hsp72 with IRE1α (Figure 5D). We were able to detect a physical interaction of endogenous Hsp72 with ectopically expressed IRE1α FL-HA as well as endogenous IRE1α in HEK 293 cells by immunoprecipitations (Figure 5E–F).

Based on the results of our immunoprecipitation experiments, we then monitored the possible effects of Hsp72 on the endoribonuclease activity of IRE1α in an in vitro assay. We have recently established an in vitro assay to monitor the endoribonuclease activity of purified IRE1α [28]. The cytosolic version of human IRE1α (recIRE1ΔN) was expressed and purified from insect cells, and then incubated with a mixture of total mRNA and ATP in the absence or presence of increasing concentrations of recombinant Hsp72. After 1 h of incubation, mRNA was re-extracted, and the cleavage of XBP1 mRNA in the splicing site was monitored by RT-PCR. As a control, actin levels were monitored. The activity of recIRE1ΔN was enhanced by the presence of recombinant Hsp72 in a dose dependent manner (Figure 5G). These results indicate that the effects of Hsp72 on IRE1α activity can be reconstituted in vitro, suggesting a direct regulation.

**Figure 6:**
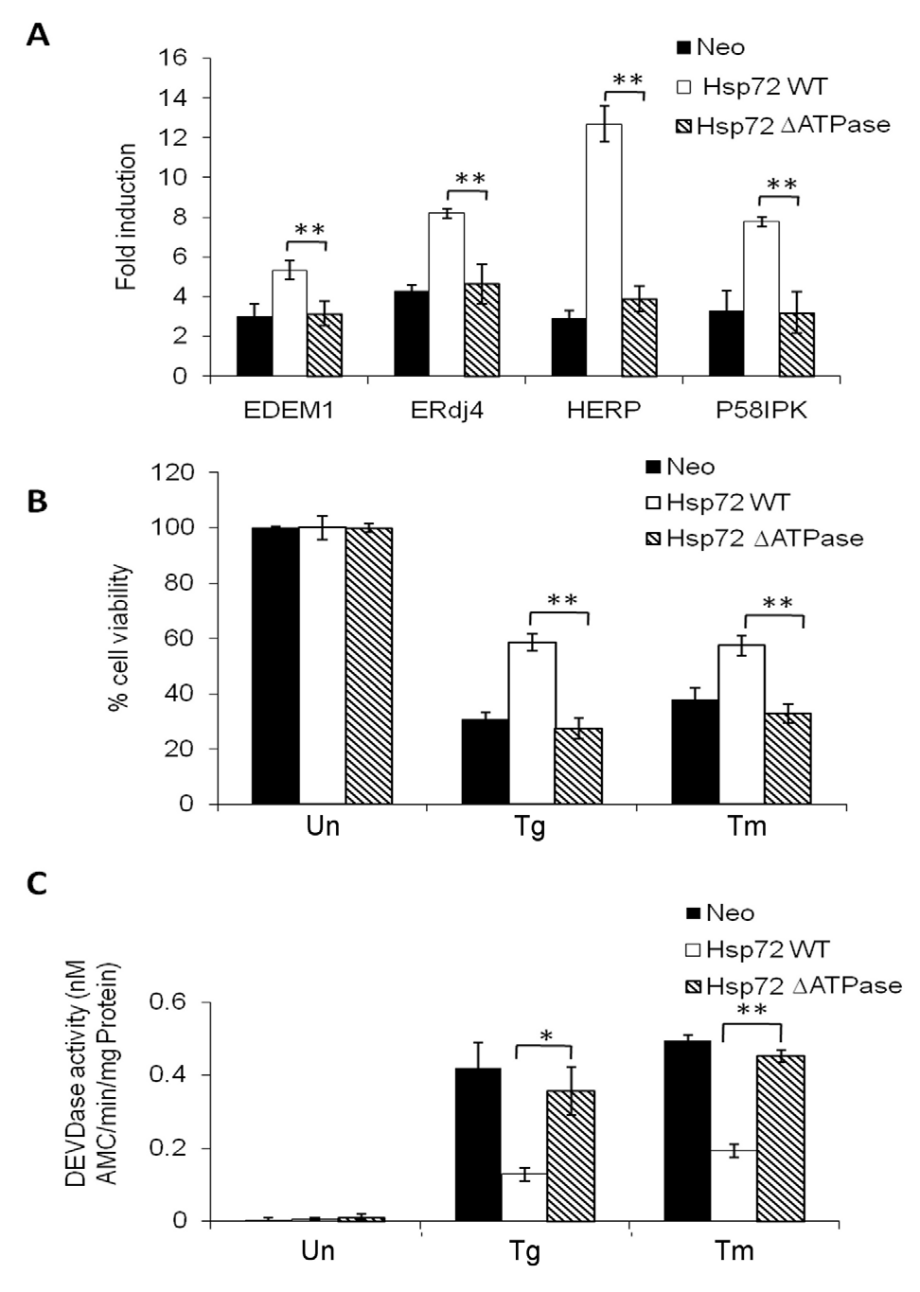
The ATPase domain of Hsp72 is necessary for activation of IRE1α/XBP1 axis and inhibition of ER stress-induced apoptosis. (A) The control (Neo), wild-type Hsp72, and ΔATPase Hsp72 expressing PC12 cells were treated with (0.25 µM) Tg for 12 h and the expression levels of indicated genes were quantified by real-time RT-PCR, normalizing against GAPDH. Average and error bars represent mean ± SD from two independent experiments performed in triplicate. ** indicates a statistical significance between Hsp72 and Hsp72 ΔATPase cells; *p*<0.005. (B) The control (Neo), wild-type Hsp72, and ΔATPase Hsp72 expressing PC12 cells were either untreated (Un) or treated (0.25 µM) Tg or (2 µg/ml) Tm for 48 h, and cell viability was determined using MTT assay. Average and error bars represent mean ± SD from three independent experiments performed in triplicate. ** indicates a statistical significance between Hsp72 and Hsp72 ΔATPase cells; *p*<0.005. (C) The control (Neo), wild-type Hsp72, and ΔATPase Hsp72 expressing PC12 cells were treated as in (B), and DEVDase activity was measured as described in Materials and Methods. Average and error bars represent mean ± SD from four independent experiments performed in duplicate. ** indicates a statistical significance between Hsp72 and Hsp72 ΔATPase cells; *p*<0.005.

The critical role of the Hsp72 ATPase domain in IRE1α binding prompted us to determine its role in ER stress-mediated IRE1α signaling. We evaluated the induction of EDEM1, ERdj4, HERP, P58^IPK^, and GRP78 in cells expressing Hsp72 or ΔATPase-Hsp72. Quantitative RT-PCR analysis revealed that induction of EDEM1, ERdj4, HERP, P58^IPK^, and GRP78 was significantly enhanced only in Hsp72 cells. Notably, induction of EDEM1, ERdj4, HERP, P58^IPK^, and GRP78 in ΔATPase-Hsp72 expressing cells was comparable to Neo cells (Figure 6A). The examination of ER stress-induced apoptosis and caspase activity in cells expressing Hsp72 or ΔATPase-Hsp72 revealed that wild-type Hsp72 expressing cells were more resistant to ER stress-induced apoptosis and caspase activation (Figure 6B–C). There was no significant difference in ER stress-induced apoptosis and caspase activation in ΔATPase-Hsp72 and Neo cells (Figure 6B–C). Collectively, these results show that the ability of Hsp72 to bind to IRE1α correlates with increased induction of UPR target genes downstream of IRE1α/XBP1 and protection against ER stress-induced apoptosis.

### Hsp72 Regulates IRE1α-XBP1 in Physiological Models

**Figure 7:**
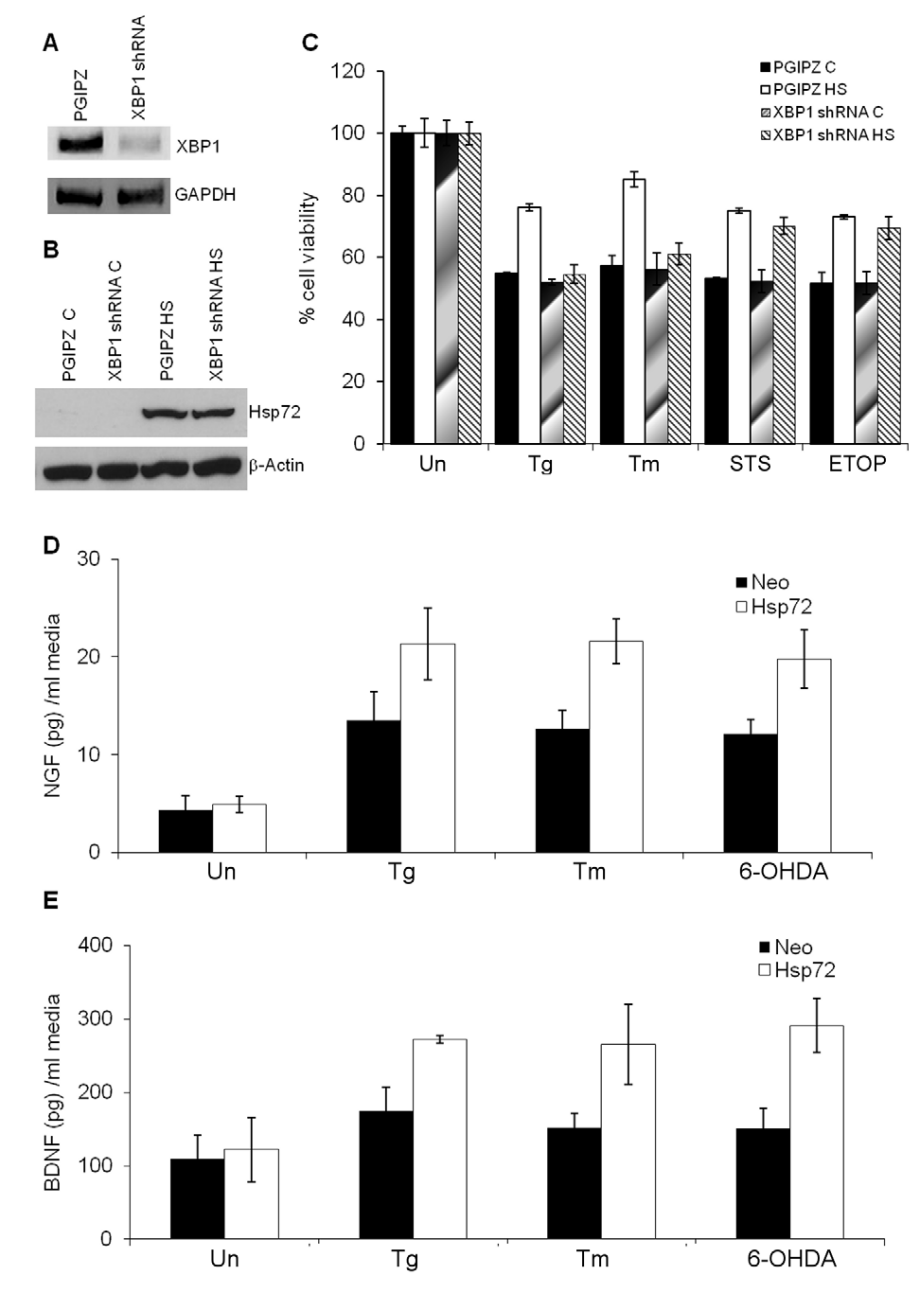
Regulation of IRE1α-XBP1 by Hsp72 contributes to thermotolerance against ER stress and increased secretion of neurotrophins. (A) PC12 cells were transduced with lentivirus expressing control non-targeting shRNA or XBP1 targeting shRNA. RT-PCR analysis of total RNA was performed to simultaneously detect unspliced XBP1 mRNA and GAPDH. The image is presented inverted for greater clarity. (B) The control (PGIPZ) or XBP1 shRNA expressing (XBP1 shRNA) PC12 cells were heat shocked for 1 h at 42°C and left to recover for 6 h. Western blots on whole cell lysates were carried out to check the expression of Hsp72 after heat shock with β-actin as loading control. (C) Normal (PGIPZ C, XBP1shRNA C) and thermotolerant (PGIPZ HS, XBP shRNA HS) control and XBP1 shRNA expressing PC12 cells were either untreated (Un) or treated with (0.25 µM) Tg for 48 h, (2 µg/ml) Tm for 48 h, (150 nM) staurosporine (STS) for 16 h, or (25 µg/ml) etoposide (ETOP) for 24 h. The reduction in cell viability was determined by MTT assay. Average and error bars represent mean ± SD from three independent experiments performed in triplicate. (D–E) The control (Neo) and Hsp72 expressing (Hsp72) PC12 cells were either untreated (Un) or treated with (0.1 µM) Tg, (0.5 µg/ml) Tm, or (50 µM) 6-OHDA for 24 h. Culture supernatant was analyzed for NGF and BDNF according to the conditions as described in Materials and Methods. Average and error bars represent mean ± SD from three independent experiments performed in triplicate. * indicates a statistical significance between Neo and Hsp72 cells; *p*<0.05. ** indicates a statistical significance between Neo and Hsp72 cells; *p*<0.005.

Mammalian cells, when exposed to a non-lethal heat shock, have the ability to acquire a transient resistance to subsequent exposures at elevated temperatures, a phenomenon termed thermotolerance. We have previously shown that mild heat shock preconditioning can induce expression of Hsp72 and protect PC12 cells against a number of cytotoxic agents [43]. To evaluate the effect of Hsp72 on IRE1α-XBP1 axis in physiological conditions, we examined the acquisition of thermotolerance in control and XBP1 knockdown PC12 cells. For this purpose parental PC12 cells were transduced with control (PGIPZ) and XBP1 targeting shRNA (XBP1 shRNA) expressing lentiviral particles. Mild heat shock preconditioning induced the expression of Hsp72 in control and XBP1 knockdown PC12 cells to comparable levels (Figure 7A–B). However, the knockdown of XBP1 specifically abrogated heat-induced acquisition of resistance against ER stress-induced apoptosis in PC12 cells (Figure 7C), but not against etoposide or staurosporine (Figure 7C). These results suggest an important role for regulation of IRE1α/XBP1 axis by Hsp72 in attainment of ER stress tolerance induced upon heat preconditioning. More importantly, these data provide evidence of a molecular crosstalk between the cytosolic heat shock response and the UPR.

The main physiological function of the XBP1 axis of the UPR is to modulate secretory pathway function, enhancing protein secretion [44],[45],[46]. PC12 is a cell line derived from a pheochromocytoma of the rat adrenal gland and secretes neurotrophins such as nerve growth factor (NGF) and brain-derived neurotrophic factor (BDNF). Therefore, we monitored the secretion of NGF and BDNF in Neo and Hsp72 cells after exposure to sublethal dose of either thapsigargin, tunicamycin, or 6-hydroxy dopamine (6-OHDA), a commonly used drug to mimic Parkinson's disease-like features in animals that also triggers ER stress [47],[48], to modulate ER physiology. First we determined the effect of Hsp72 expression on 6-OHDA-induced death in PC12 cells. We found that Hsp72 cells were resistant to 6-OHDA induced death as compared to Neo cells (Figure S1). In addition secretion of NGF and BDNF into the cell-culture media of Hsp72 cells was more after treatment with thapsigargin, tunicamycin, and 6-OHDA, than in media of Neo cells (Figure 7D–E). These data indicate that Hsp72 regulates secretion of neurotrophins (NGF and BDNF) by PC12 cells most likely mediated by the modulation of IRE1α/XBP1 function.

## Discussion

Previous studies have shown that Hsp72 overexpression protects cells from death induced by inhibiting multiple cell death pathways, including in models of hypoxia and ischemia/reperfusion [3]. Although the anti-apoptotic effects of Hsp72 have been noted in several systems, the molecular mechanisms that mediate this effect are largely unclear. In the present study, we revealed a new function of Hsp72 as a critical regulator of the UPR and adaptation to ER stress conditions. Chronic ER stress signals converge into the mitochondrial intrinsic death pathway that involves release of cytochrome *c*, Apaf-1, formation of apoptosome, and activation of caspase proteases [19],[39],[49],[50]. The interaction of Apaf-1 with cytochrome *c* and ATP, leading to activation of caspase-9, has been shown to be inhibited by Hsp72 in in vitro conditions [7],[8]. Our results suggest that Hsp72 can prevent cytochrome *c* release from the mitochondria and that the reported ability of Hsp72 to block caspase-9 activation in the cytosolic fraction is possibly due to the high salt concentration in the Hsp72 preparation [51]. In this study we describe a new function of Hsp72 where it acts upstream of MOMP by controlling adaptive responses against ER stress, enhancing cell survival. These effects were due to a direct interaction between Hsp72 and the UPR stress sensor IRE1α, possibly controlling IRE1α's activity. Hsp72 has been reported to inhibit CHOP- and TNFα-induced apoptosis by binding to BAX and preventing its translocation to mitochondria [10]. Furthermore, the ATPase domain of Hsp72 is required for inhibition of CHOP- and TNFα-induced apoptosis [10]. Consistent with this, our results show that an ATPase domain deletion mutant of Hsp72 was unable to protect cells against ER stress-induced apoptosis. In contrast, the ATPase domain was dispensable for inhibition of apoptosis induced by oxygen-glucose deprivation [52], serum withdrawal, staurosporine [53], or heat [54], suggesting different mechanisms of action.

There are a number of physiological and pathological conditions where concomitant induction of Hsp72 expression and ER stress has been reported. First, 6-OHDA induces ER stress and upregulation of Hsp72 in cellular models of Parkinson's disease [47],[55],[56]. Second, proteasome inhibitors have been shown to increase the expression of Hsp72 [57] and to induce ER stress and UPR [58]. Third, Hsp72 expression is enhanced in cancer cells harboring mutant p53 due to derepression of Hsp72 promoter and Hsp72 has been reported to be overexpressed in many cancers [59]. Activation of the UPR is an adaptive response that allows cells to survive prolonged ER stress/hypoxia conditions in solid tumors [60],[61]. In light of our results we speculate that co-activation of Hsp72 and the UPR may represent a mechanism for the fine-tuning of IRE1α, providing a functional crosstalk between both stress pathways.

What is the biological significance of the ability of Hsp72 to modulate the UPR by binding to IRE1α? Transcriptional activation of target genes that enhance ER protein-folding capacity and degradation of misfolded ER proteins plays an important role in cytoprotective function of XBP1[17]. Peter Walter's group has shown that XBP1 mRNA splicing levels decline after prolonged ER stress, whereas PERK signaling is sustained over time [62]. Inactivation of XBP1 splicing was proposed to sensitize cells to cell death after chronic or irreversible ER stress. Similarly, knocking down XBP1 or IRE1α enhances cell death under conditions of chronic ER stress [28]. Further, experimental prolonging of IRE1α signaling independent of ER stress can promote cell survival [41],[62]. Here we demonstrate that IRE1α/XBP1 signaling is specifically enhanced by Hsp72 expression. Our data show that Hsp72 can enhance the amplitude of IRE1α signaling, delaying the inactivation phase of XBP1 mRNA splicing. These effects were functionally linked to the ability of Hsp72 to augment cell survival under conditions of ER stress. In addition to pathological conditions related to chronic or irreversible ER stress, the UPR plays a central role in physiological conditions associated with protein folding stress due to high demand for protein folding [25]. A key role of XBP1 has been proposed in vivo in different secretory cells including B cells, exocrine pancreas, and salivary glands [44],[45],[46], where activation of XBP1-transcriptional responses enhances secretion, improving cell survival. In agreement with this idea, we observed that Hsp72 expression enhances secretion of neurotrophins in PC12 dopaminergic cells (Figure 7). Our results confirm the notion that attenuation of IRE1α signaling during chronic ER stress is a key step in cell fate determination after induction of UPR. It has been shown that UPR induction can lead to proteolytic cleavage of IRE1α, releasing fragments containing the kinase and nuclease domains that accumulate in the nucleus [63]. However, we did not observe any change in subcellular localization of IRE1α during the UPR in Neo and Hsp72 cells (Figure S2). Furthermore Hsp90 has been shown to increase the half-life of IRE1α and PERK by binding to their cytoplasmic domains [64]. Hsp72 expression had no effect on the half-life of IRE1α (Figure S3).

The ER-lumenal domain of PERK, IRE1α, and ATF6 interacts with the ER chaperone GRP78, however upon accumulation of unfolded proteins GRP78 dissociates from these molecules, leading to their activation [17]. Although ATF6, PERK, and IRE1α share functionally similar luminal sensing domains and are activated in cells treated with ER stress inducers in vitro, they are selectively activated in vivo by the physiological stress of unfolded proteins [17],[18]. These differences may explain the different kinetics in the activation of IRE1α, PERK, and ATF6 in response to various ER stress inducers. However, the differences in terms of tissue-specific regulation of the UPR in vivo may be explained by the formation of unique protein complexes through association of adaptor and modulator proteins. It has been proposed that a complex protein platform, known as the UPRosome [24],[25], operates at the ER membrane to control IRE1α activity. Our results identify Hsp72 as a new component of this UPRosome where binding of Hsp72 to IRE1α enhances IRE1α-XBP1 signaling at the ER and inhibits ER stress-induced apoptosis. Our results suggest that Hsp72 can bind to the monomeric and nonphosphorylated cytoplasmic tail of IRE1α. Furthermore the interaction of Hsp72 and IRE1α is not affected by ER stress mediated phosphorylation and oligomerization of IRE1α. What is the molecular mechanism by which Hsp72 stimulates the RNase activity of IRE1α? There are several possibilities by which Hsp72 might regulate RNase activity of IRE1α. Hsp72 may regulate IRE1α either by allosteric interactions or by altering the binding of other regulatory proteins (BAX, BAK, BI-1, AIP1, and RACK) to IRE1α. Our results showing that recombinant Hsp72 can enhance the RNase activity of purified IRE1α in in vitro conditions suggest an allosteric mechanism (Figure 5G). However it is possible that in a cellular context other mechanisms such as altering the binding of other regulatory proteins (BAX, BAK, BI-1, AIP1, and RACK) to IRE1α are also involved. Previous reports and our data collectively support a model in which a fine balance of anti- and pro-apoptotic proteins at the ER membrane modulates the amplitude of IRE1α signaling, thereby regulating the cellular sensitivity to ER stress conditions.

## Materials and Methods

### Cell Culture and Treatment

Rat pheochromocytoma PC12 cells (obtained from ECACC) were cultured in Dulbecco's modified Eagle's medium (DMEM) from Sigma (D6429) supplemented with 10% heat inactivated horse serum, 5% foetal bovine serum, and 1% penicillin/streptomycin (Sigma) at 37°C, 5% CO_2_ in humidified incubator. Appropriate number of cells was seeded 24 h prior to treatment. To induce apoptosis, cells were treated with 0.25 µM thapsigargin, 2 µg/ml tunicamycin, 150 nM staurosporine, or 25 µg/ml etoposide for the indicated time periods. Stock solutions of 6-Hydroxydopamine were made freshly in sodium metabisulfite (1 M) prior to experiment. PC12 cells were treated with 200 µM 6-OHDA for 24 h before analysis. All reagents were from Sigma-Aldrich unless otherwise stated.

### Plasmids and Transfection

The plasmid expressing wild type Hsp72, ΔATPase-Hsp72, or ΔEEVD-Hsp72 under the CMV promoter were kind gifts from Dr. Tomomi Gotoh, Kumamoto University, Japan [10]. The expression vector for wild type IRE1α, IRE1α KA, IRE1α ΔC, and IRE1α ΔRNase under the CMV promoter were kind gifts from Dr. Kazunori Imaizumi, University of Miyazaki, Japan [65] and expression plasmids for wild type IRE1α-HA or IRE1α ΔC-HA are reported previously [28]. The plasmids containing shRNAs targeting rat XBP1 were obtained from GeneCopoeia, Rockville, USA (RSH045024-HIV U6). Transfections of cells were carried out using Lipofectamine 2000 (Invitrogen) according to the manufacturer's protocol.

### Cell Viability Assay

Viability of cells after treatment was analyzed by MTT assay. After 48 h of treatment, 1 mg/ml concentration of MTT ((3-(4, 5-dimethylthiazol-2-yl)-2, 5-diphenyl tetrazonium bromide) was added to the wells and incubated at 37°C for 3 h. The reaction was stopped with a stop mix containing 20% SDS in 40% dimethyl formamide. The color intensity is measured at 550 nm and percentage cell viability is calculated using the untreated samples as 100%.

### Annexin V Staining

Externalization of phosphatidylserine (PS) to the outer leaflet of the plasma membrane of apoptotic cells was assessed with annexin V-FITC as described earlier [66]. Briefly, cells were collected by centrifugation at 350 g, washed once in ice-cold calcium buffer (10 mM HEPES/NaOH, pH 7.4, 140 mM NaCl, 2.5 mM CaCl_2_), and incubated with annexin V-FITC or with annexin V-PE for 15 min on ice. Prior to analysis 300 µl of binding buffer containing 4 µl of PI (50 µg/ml) was added and analyzed on a FACSCalibur flow cytometer (Becton Dickinson).

### Analysis of DEVDase Activity

Cells were harvested and pelleted by centrifugation at 350 g. After washing in PBS, cell pellets were re-suspended in 50 µl of PBS and 25 µl was transferred to duplicate wells of a microtiter plate and snap-frozen in liquid nitrogen. To initiate the reaction, 50 µM of the caspase substrate carbobenzoxy-Asp-Glu-Val-Asp-7-amino-4-methyl-coumarin (DEVD-AMC, Peptide Institute Inc.) in assay buffer (100 mM HEPES, pH 7.5, 10% sucrose, 0.1% CHAPS, 5 mM DTT and 0.0001% Igepal-630, pH 7.25) was added to cell lysates. Liberated free AMC was measured by a Wallac Victor 1420 Multilabel counter (Perkin Elmer Life Sciences) using 355 nm excitation and 460 nm emission wavelengths at 37°C at 60 s intervals for 25 cycles. The data were analyzed by linear regression and enzyme activity was expressed as nM of AMC released × min^−1^×mg^−1^ total cellular protein.

### Measurement of ΔΨ_m_

Mitochondrial transmembrane potential was determined by using the fluorescent probe tetramethylrhodamine ethyl ester (TMRE, Molecular Probes) as previously described [67]. Briefly, cells were trypsinized and incubated with TMRE at RT for 30 min in the dark and analyzed by flow cytometry using a FACSCalibur instrument.

### RNA Extraction, RT-PCR, and Real Time RT-PCR

Total RNA was isolated using RNeasy kit (Qiagen) according to the manufacturer's instructions. Reverse transcription (RT) was carried out with 2 µg RNA and Oligo dT (Invitrogen) using 20 U Superscript II Reverse Transcriptase (Invitrogen). The cDNA product was subjected to 25–35 cycles of PCR using the forward primer 5-TTACGAGAGAAAACTCATGGGC-3 and reverse primer 5-GGGTCCAACTTGTCCAGAATGC-3 specific for Rat XBP1. GAPDH (forward: ACCACAGTCCATGCCATC; reverse: TCCACCACCTGTTGCTG) was used as an endogenous control. Real-time PCR method to determine the induction of UPR target genes has been described previously[68]. Briefly, cDNA products were mixed with 2× TaqMan master mixes and 20× TaqMan Gene Expression Assays (Applied Biosystems) and subjected to 40 cycles of PCR in StepOnePlus instrument (Applied Biosystems). Relative expression was evaluated with ΔΔC_T_ method.

### Preparation of Cytosolic Extracts

The cells were washed in ice-cold PBS and lysed using cell lysis and mitochondrial intact buffer (CLAMI) containing 250 mM sucrose, 70 mM KCl dissolved in 1× PBS with 0.5 mM DTT and 2.5 µg/ml Pepstatin and 0.2 g/ml digitonin. The cells were allowed to swell on ice for 5 min. The cell suspension was centrifuged at 400 g for 5 min and the pellet was removed. The supernatant was transferred to a clean eppendorf tube and the mitochondrial and microsomal fractions were separated by spinning at 20,000 g for 5 min. The cytosolic fraction was removed and prepared for Western blot by adding 5× sample buffer.

### Western Blotting

Cells were washed once in ice-cold PBS and lysed in whole cell lysis buffer (20 mM HEPES pH 7.5, 350 mM NaCl, 0.5 mM EDTA, 1 mM MgCl_2_, 0.1 mM EGTA, and 1% NP-40) after stipulated time of treatments and boiled at 95°C with Laemmli's SDS-PAGE sample buffer for 5 min. Protein concentration was determined by Bradford method. Equal amounts (20 µg/lane) of protein samples were run on an SDS polyacrylamide gel. The proteins were transferred onto nitrocellulose membrane and blocked with 5% milk in PBS-0.05% Tween. The membrane was incubated with the primary antibody Hsp72 (Stressgen SPA-810), Caspase-3 (Cell Signaling Technology, Cat# 9662), Cytochrome *c* (BD Pharmingen, Cat# 556433), XBP1 (Santa Cruz Biotechnology, Inc, Cat# sc-7160), CHOP (Santa Cruz Biotechnology, Inc, Cat# sc-793), phosphorylated eIF2α (Cell Signaling Technology, Cat# 3597), total eIF2α (Cell Signaling Technology, Cat# 2103), phosphorylated JNK (Cell Signaling, Cat# 9255S), IRE1α (Cell Signaling Technology, Cat# 3294S), or β-Actin (Sigma, Cat# A-5060) for 2 h at room temperature or overnight at 4°C. The membrane was washed 3 times with PBS-0.05% Tween and further incubated in appropriate horseradish peroxidase-conjugated secondary antibody (Pierce) for 90 min. Signals were detected using West pico chemiluminescent substrate (Pierce).

### Immunoprecipitations

Immunoprecipitation of HA-tagged wild-type IRE1α or IRE1α ΔC was performed using Pierce Profound mammalian HA tagged IP/Co-IP kit (23615). Briefly, cell lysates were incubated with HA-agarose slurry in IP column overnight. Agarose beads were washed twice with TBS containing 0.05% Tween. Protein complexes were extracted by boiling the beads with 2× lane marker buffer and analyzed by Western blotting as described above. For immunoprecipitation of Hsp72, cleared protein extracts were incubated with anti-Hsp72 polyclonal antibody (Stressgen SPA-811) overnight at 4°C, followed by 100 µl of a 12% suspension of protein A-Sepharose for 1 h at 4°C, and then washed three times with TBS-0.05% Tween. Protein complexes were eluted by boiling in 2× lane marker buffer and analyzed by Western blotting as described above.

### In Vitro IRE1α Activity Assays

The effect of Hsp72 on activity of IREα was monitored using recombinant human IRE1αΔN-HIS produced as GST fusion protein using the Prescission Protease cleaved system. IRE1α ΔN was incubated with recombinant Hsp70 (Stressgene) in a total volume of 50 µl for 1 h at 30°C with 10 µg of total mRNA as substrate (obtained from mouse brain cortex because of minimal basal levels of spliced XBP1 mRNA) in a buffer containing 20 mM HEPES (pH 7.3), 1 mM DTT, 10 mM magnesium acetate, 50 mM potassium acetate, and 2 mM ATP. Then, mRNA was re-extracted with 500 µl of Trizol, and the endoribonuclease activity of IRE1α was monitored by RT-PCR using the XBP1 mRNA splicing assay that employs a set of primers that closely surround the processing site. Using this method, we observed a decrease in the amount of nonspliced XBP1 mRNA due to its cleavage by IRE1αN-HIS as we previously described [28].

### Knockdown of XBP1

We generated stable subclones of PC12-Hsp72 with reduced levels of XBP1 by targeting XBP1 mRNA with shRNA using the lentiviral expression vector psiHIV-U6 (GeneCopoeia). The targeting sequences identified for rat XBP1 were XBP1 shRNA1: 5-actgcgcgagatagaaaga-3; XBP1 shRNA2: 5- gttgcctcttcagattctg-3; XBP1 shRNA3: 5-gagagccaaactaatgtgg-3; and XBP1 shRNA4: 5-ctgaggtcttcaaaggtat-3.

### Heat Preconditioning of PC12 Cells

Cells were seeded in T25 flasks 24 h prior to heat preconditioning. The flasks were sealed with parafilm and immersed in water bath set at 42°C for 1 h. The cells were left to recover for 6 h at 37°C before treating with apoptosis inducing agents. Media was changed prior to treatment.

### ELISA for BDNF and NGF Release

Cells were treated with 0.1 µM Tg or 0.5 µg/ml Tm or 50 µM 6-OHDA for 24 h to induce UPR. Culture media was analyzed for NGF or BDNF release using β-NGF (DY 256) or BDNF (DY 248) DuoSet ELISA development kit according to manufacturer's protocol (R&D Systems). The amount of NGF or BDNF released into the media was calculated using the standard curve generated in parallel with recombinant NGF and BDNF.

### Statistical Analysis

All the experiments were repeated at least 2 times. Results are expressed as mean ± standard deviation. Statistical analyses of the results were done with Student's *t* test using Graphpad (http://www.graphpad.com).

## Supporting Information

Figure S1 **Overexpression of Hsp72 protects PC12 cells from 6-OHDA treatment induced cell death.** The control (Neo) and Hsp72 expressing (Hsp72) PC12 cells were either untreated (Un) or treated with 200 mM 6-OHDA for 24 h. Reduction in cell viability was analyzed by sub G1 peak. Average and error bars represent mean ± SD from three independent experiments performed in triplicates.
(0.06 MB TIF)

Figure S2 **Subcellular localization of IRE1α is not affected by Hsp72. Hsp72 and Neo expressing cells were transfected with IRE1α-GFP.** After 24 h of treatment with Tg, cells were fixed in 3.7% formaldehyde and the coverslips were mounted using Vectashield mounting medium with DAPI (H-1200). Cells were visualized using a Nikon microscope fitted with appropriate filters.
(0.56 MB TIF)

Figure S3 **Overexpression of Hsp72 does not affect the half-life of IRE1.** (A) Control (Neo) and Hsp72 expressing (Hsp72) PC12 cells were treated with (10 ng/ml) Tg for the indicated time. Immunoblotting of total protein was performed using antibodies against IRE1α and β-actin. (B) In the experiment described in (A), band density of IRE1α was calculated by densitometry and normalized against β-actin. Average and error bars represent mean ± SD from two independent experiments.
(0.17 MB TIF)

## Abbreviations

6-OHDA: 6-hydroxy dopamine
AIF: apoptosis-inducing factor
AIP1: ASK1-interacting protein 1
ALS: Amyotrophic Lateral Sclerosis
ATF6: activating transcription factor 6
BDNF: brain-derived neurotrophic factor
BI-1: Bax inhibitor-1
DMEM: Dulbecco's modified Eagle's medium
ER: endoplasmic reticulum
ERN1: endoplasmic reticulum-to-nucleus signaling 1
IRE1α: inositol requiring 1
NGF: nerve growth factor
PERK: double-stranded RNA-activated protein kinase (PKR)-like ER kinase
PTP 1B: Protein Tyrosine Phosphatase 1B
RT: reverse transcription
UPR: unfolded protein response
XBP1: X-box binding protein
XBP1u: unspliced XBP1
