# Supplementary figures and images for: HSP72 Protects Cells from ER Stress-induced Apoptosis via Enhancement of IRE1α-XBP1 Signaling through a Physical Interaction
Source: PLoS Biol. 2010 Jul 6;8(7):e1000410. doi: 10.1371/journal.pbio.1000410 (PMC2897763; doi:10.1371/journal.pbio.1000410)

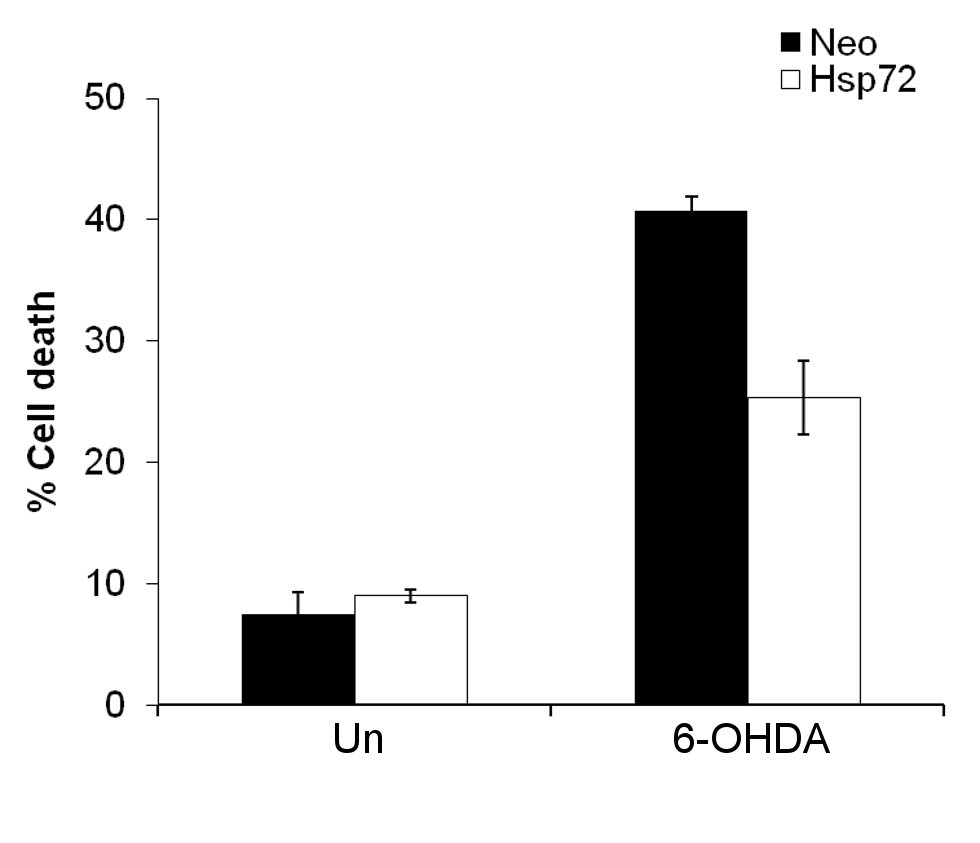

Supplement: Figure S1 — Overexpression of Hsp72 protects PC12 cells from 6-OHDA treatment induced cell death. The control (Neo) and Hsp72 expressing (Hsp72) PC12 cells were either untreated (Un) or treated with 200 mM 6-OHDA for 24 h. Reduction in cell viability was analyzed by sub G1 peak. Average and error bars represent mean ± SD from three independent experiments performed in triplicates. (0.06 MB TIF) [file pbio.1000410.s001.tif]

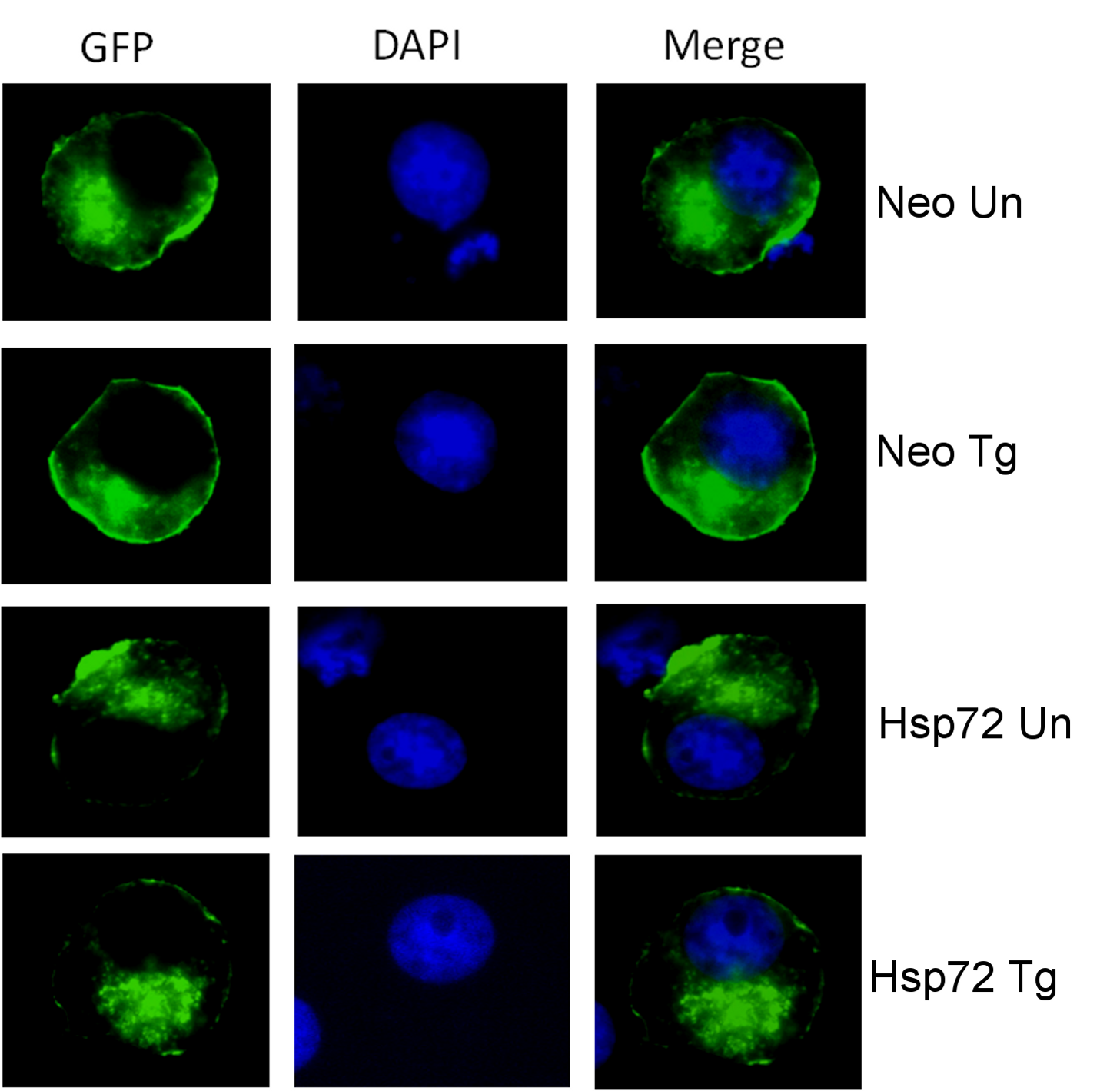

Supplement: Figure S2 — Subcellular localization of IRE1α is not affected by Hsp72. Hsp72 and Neo expressing cells were transfected with IRE1α-GFP. After 24 h of treatment with Tg, cells were fixed in 3.7% formaldehyde and the coverslips were mounted using Vectashield mounting medium with DAPI (H-1200). Cells were visualized using a Nikon microscope fitted with appropriate filters. (0.56 MB TIF) [file pbio.1000410.s002.tif]

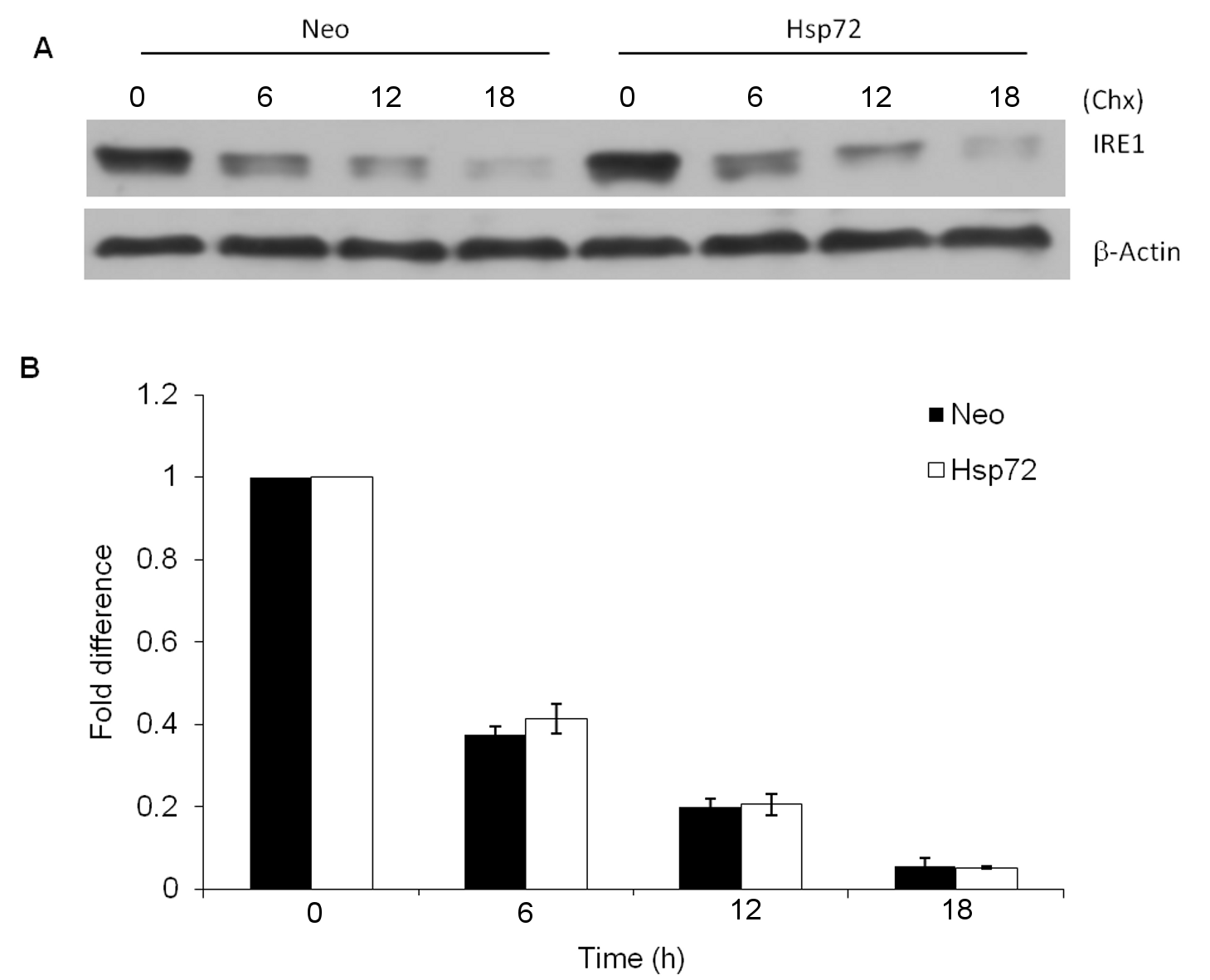

Supplement: Figure S3 — Overexpression of Hsp72 does not affect the half-life of IRE1. (A) Control (Neo) and Hsp72 expressing (Hsp72) PC12 cells were treated with (10 ng/ml) Tg for the indicated time. Immunoblotting of total protein was performed using antibodies against IRE1α and β-actin. (B) In the experiment described in (A), band density of IRE1α was calculated by densitometry and normalized against β-actin. Average and error bars represent mean ± SD from two independent experiments. (0.17 MB TIF) [file pbio.1000410.s003.tif]
